# Visualizing the actin cytoskeleton in living plant cells using a photo-convertible mEos::FABD-mTn fluorescent fusion protein

**DOI:** 10.1186/1746-4811-4-21

**Published:** 2008-09-19

**Authors:** Mike Schenkel, Alison M Sinclair, Daniel Johnstone, J Derek Bewley, Jaideep Mathur

**Affiliations:** 1 Department of Molecular and Cellular Biology, University of Guelph, Guelph, Ontario, N1G 2W1, Canada

## Abstract

**Background:**

The actin cytoskeleton responds quickly to diverse stimuli and plays numerous roles in cellular signalling, organelle motility and subcellular compartmentation during plant growth and development. Molecular and cell biological tools that can facilitate visualization of actin organization and dynamics in a minimally invasive manner are essential for understanding this fundamental component of the living cell.

**Results:**

A novel, monomeric (m) Eos-fluorescent protein derived from the coral *Lobophyllia hemprichii *was assessed for its green to red photo-convertibility in plant cells by creating mEosFP-cytosolic. mEosFP was fused to the F-(filamentous)-Actin Binding Domain of the mammalian Talin gene to create mEosFP::FABDmTalin. Photo-conversion, visualization and colour quantification protocols were developed for EosFP targeted to the F-actin cytoskeleton. Rapid photo-conversion in the entire cell or in a region of interest was easily achieved upon illumination with an approximately 400 nm wavelength light beam using an epi-fluorescent microscope. Dual color imaging after photo-conversion was carried out using a confocal laser-scanning microscope. Time-lapse imaging revealed that although photo-conversion of single mEosFP molecules can be rapid in terms of live-cell imaging it involves a progressive enrichment of red fluorescent molecules over green species. The fluorescence of photo-converted cells thus progresses through intermediate shades ranging from green to red. The time taken for complete conversion to red fluorescence depends on protein expression level within a cell and the quality of the focusing lens used to deliver the illuminating beam. Three easily applicable methods for obtaining information on fluorescent intensity and colour are provided as a means of ensuring experimental repeatability and data quantification, when using mEosFP and similar photo-convertible proteins.

**Conclusion:**

The mEosFP::FABD-mTn probe retains all the imaging qualities associated with the well tested GFP::mTn probe while allowing for non-invasive, regional photo-conversion that allows colour based discrimination within a living cell. Whereas a number of precautions should be exercised in dealing with photo-convertible probes, mEosFP::FABD-mTn is a versatile live imaging tool for dissecting the organization and activity of the actin cytoskeleton in plants.

## Background

Since the cloning of the Green Fluorescent Protein (GFP) from *Aequorea victoria *[1] and its successful expression in living cells [2], fluorescent proteins have become integral components of the biologists' tool kit for understanding subcellular dynamics and interactions [3,4]. Multicoloured fluorescent proteins are now available from a variety of marine organisms [5,6]. The availability of varicoloured fluorescent proteins has spawned diverse strategies to target them to different sub-compartments and components of the cell. In plant research subcellular-targeted fluorescent proteins have been used to illuminate the micro-world of the plant cell and made major contributions to our understanding of plants (reviewed in [7]). A recently created web-resource provides a list of subcellular-targeted fluorescent probes developed over the last decade for plants .

The cytoskeleton forms a vital component of all living cells. In plants both the actin and microtubule cytoskeletons play pivotal roles in inter- and intra-cellular signaling, cell compartmentalization and subcellular trafficking. In recent years, the actin cytoskeleton has emerged as a major player in plant cell growth and morphogenesis [8-10]. The organization of polymerized actin filaments (F-actin) in a cell has major influence on local growth and differentiation of a cell [11,12]. While fluoro-chrome-linked phallotoxins and immuno-reagents can be used to observe the actin cytoskeleton in fixed plant cells, recent trends favor live-imaging of the dynamic actin cytoskeleton. Two fusion proteins have become widely accepted for visualizing F-actin in living cells: These are the GFP::mTn [13] and the GFP::ABD2-FIMBRIN1 [14] constructs. The former probe is composed of a modified GFP fused to the F-actin binding C-terminal 197 amino acids of the Talin gene from mouse [13]. The latter probe [14-16] employs the second actin-binding domain (ABD2) from an actin bundling protein FIMBRIN1 [17,18]. In addition to the above, an inducible GFP::mTn probe under the control of an ethanol-inducible promoter has been created [19], and Wang et al. [20] have further modified the GFP::FIM1-ABD2 probe by fusing GFP to both the N- and C- termini of ABD2 (35S-GFP::ABD2::GFP). Each probe for visualizing F-actin in plants has its benefits and drawbacks (discussed in [13,19-21]. Many of the artifacts reported might be attributed to transient over-expression [19] or increased probe stability that interferes with actin dynamics [20]. While recent trends appear to prefer the xFP::FIM1-ABD2 fusion protein over the xFP::mTn probe, in our hands both probes can perform equally well provided care is taken to maintain certain baseline growth and development conditions. Transgenic lines for both probes when stressed do display many of the artifacts highlighted in the studies of Ketelaar et al. [19] and Holweg [21] and underscore the importance of rigorous observations in reporting changes in the actin cytoskeleton. Though the fluorescence intensity between different transgenic lines and cell types expressing either probe can vary considerably, an F-actin labeling pattern that might be considered distinctive for either probe has not been observed in different cell types (Mathur, unpublished). Nevertheless, their perceived limitations and advantages aside, the availability of two independent probes highlighting F-actin is a useful development for plant biologists because it provides cross-checks on observations and thus lends greater credibility to studies on the actin cytoskeleton in plant cells.

Both GFP::mTn and GFP::FIM1-ABD2 probes have certain limitations; they highlight F-actin within a cell in a single fluorescent colour. Regional changes in F-actin organization that may occur are thus difficult to discriminate and thus local interactions of actin with other organelles cannot be easily captured. Considering that the myriad roles of the actin cytoskeleton depend upon its dynamic nature and local alterations and adjustments in F-actin arrays [12] it is important to have locally-inducible F-actin probes. Although an ethanol-inducible GFP::mTn probe [19] has been created it is difficult to control the degree of its over-expression since neither the uptake of the inducing chemical, nor its degradation and local concentration-dependent effects can be controlled at the cellular level. As demonstrated by Ketelaar et al. [19] the massive flooding of a cell with the GFP::mTn fusion protein through transient over-expression leads to aberrant F-actin structures and consequent developmental abnormalities in the affected cells.

Recently, a number of photo-activable fluorescent proteins such as PA-GFP and [22], photo-convertible FPs such as Kaede and Dendra, and photo-switchable FPs such as PS-CFP2, [23] KindlingFP and Dronpa have become available [6,24]. The newly discovered proteins change their fluorescent properties radically in response to relatively mild irradiation. Through local photo-activation or -deactivation of fluorescence, these probes permit specific highlighting of a subset of subcellular targets and thus raise the level of precision [6]. PA-GFP fused to a calnexin membrane-binding domain has been used to locally highlight the ER [25], whereas KaedeFP has been targeted to mitochondria, peroxisomes and chloroplasts [26].

EosFP is a photo-convertible protein derived from the scleractinian coral *Lobophyllia hemprichii*. Its fluorescence changes irreversibly from green (emission max. 516 nm) to red (emission max. 581 nm) upon illumination with a wavelength of approximately 400 nm [27]. Although the wild-type EosFP is tetrameric it has been engineered to yield dimeric, monomeric and tandem-dimeric forms while maintaining a high photo-convertibility and fluorescence quantum yield [27]. Recently EosFP has been utilized for demonstrating that clathrin-dependent endocytosis in plants is the predominant pathway for the internalization of numerous plasma-membrane-resident proteins including PIN auxin efflux carriers [28]. A photo-inducible probe for visualizing the actin cytoskeleton in plants has not been reported using any of the optical highlighter proteins.

We have used monomeric EosFP to create a novel photo-convertible mEosFP::FABD-mTn probe for highlighting F-actin in plants. The method for its efficient photo-conversion in living plant cells using epi-fluorescent and confocal laser scanning microscopes is presented.

## Results and discussion

### Efficient photo-conversion of mEosFP-cytosolic in plant cells

**Figure 1:**
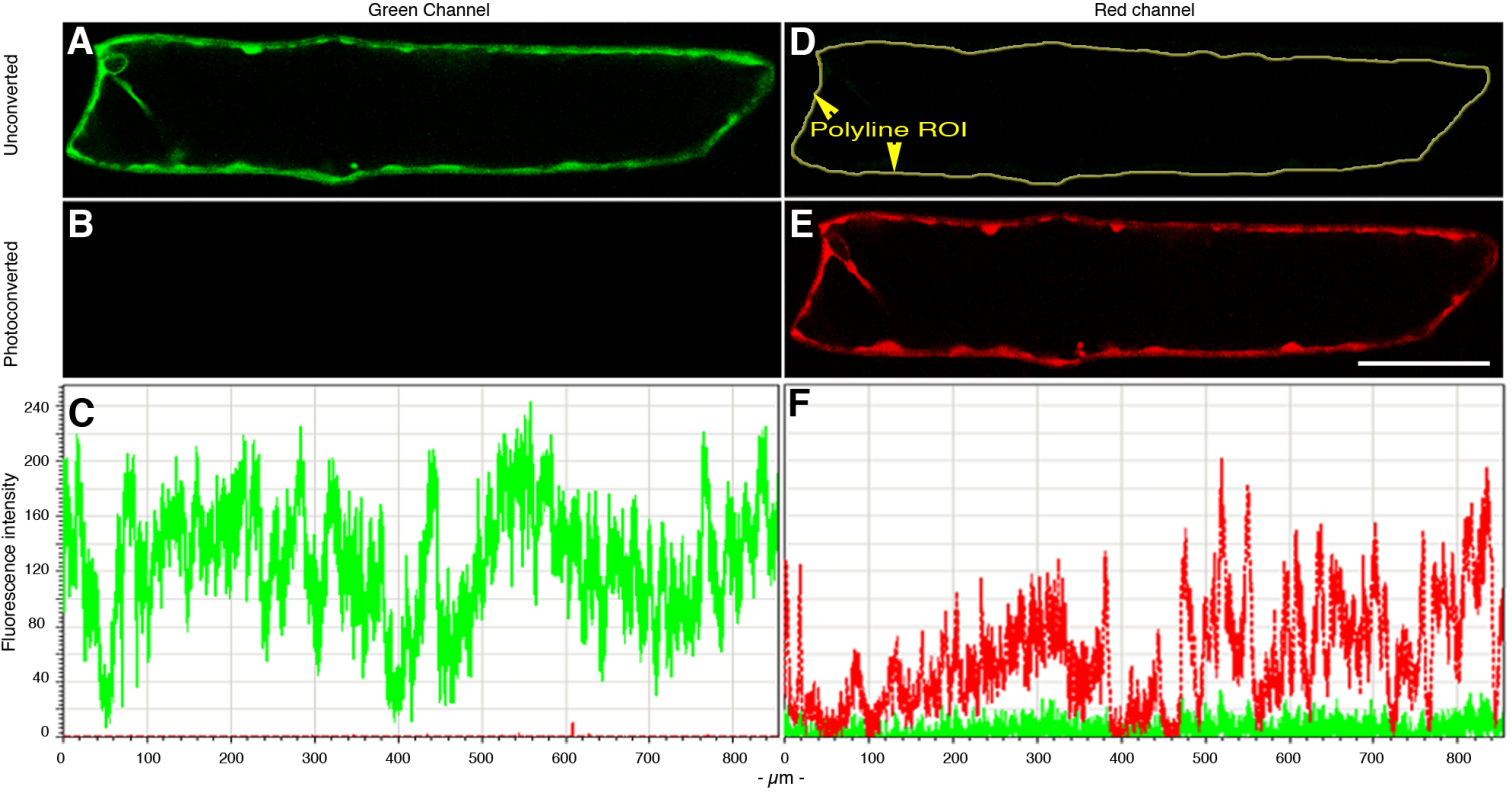
**Photo-conversion of mEosFP-cytosolic following its transient expression in an onion epidermal cell**. A.B.C. Unconverted mEosFP-Cyto visualized in green channel (500 to 525 nm) 'A'; and red channel (585 to 680 nm) 'B'. C depicts the fluorescence intensity graph observed in images A and B using the poly-line ROI (arrowheads) traced out in panel D. D.E.F. mEosFP-cyto fluorescence in green 'D' and red 'E' channels observed approximately 10 seconds after photo-conversion. F depicts the combined fluorescence intensity for D and E. Images were acquired within 10 seconds of photo-conversion. Note the shift in fluorescence intensities of red and green fluorescence between pre- and post-conversion states.

mEosFP, kindly provided by Dr. J. Wiedenmann (Ulm, Germany) was cloned into the plant expression vector pCAMBIA 1300  under a Cauliflower Mosaic Virus 35S promoter to yield mEosFP-cytosolic. Biolistic bombardment of onion epidermal cells with 1 μm diameter gold particles coated with the mEosFP-cytosolic DNA resulted in cells that fluoresced green upon illumination through an Endow GFP-LP filter cube (Figure 1A). The same cells visualized using a TRITC filter did not exhibit any fluorescence (Figure 1B, C). When the cells were exposed to ca. 350 ± 50 nm wavelength for 5–10 seconds their green fluorescence was rapidly lost (Fig. 1D) as they converted into bright red fluorescent cells (Figure 1E, F). A region of interest (ROI) that outlined the cell perimeter (arrowheads – Figure 1D) was created using the poly-line function in proprietary Leica TCS-SP5 software and produced fluorescence intensity histograms for the green and red acquisition channels (Figure 1C, F). The histograms underscore the efficient photo-conversion of mEosFP. The fluorescent protein was developed further for highlighting F-actin in plant cells.

### mEosFP::FABD-mTn highlights F-actin in green and red

**Figure 2:**
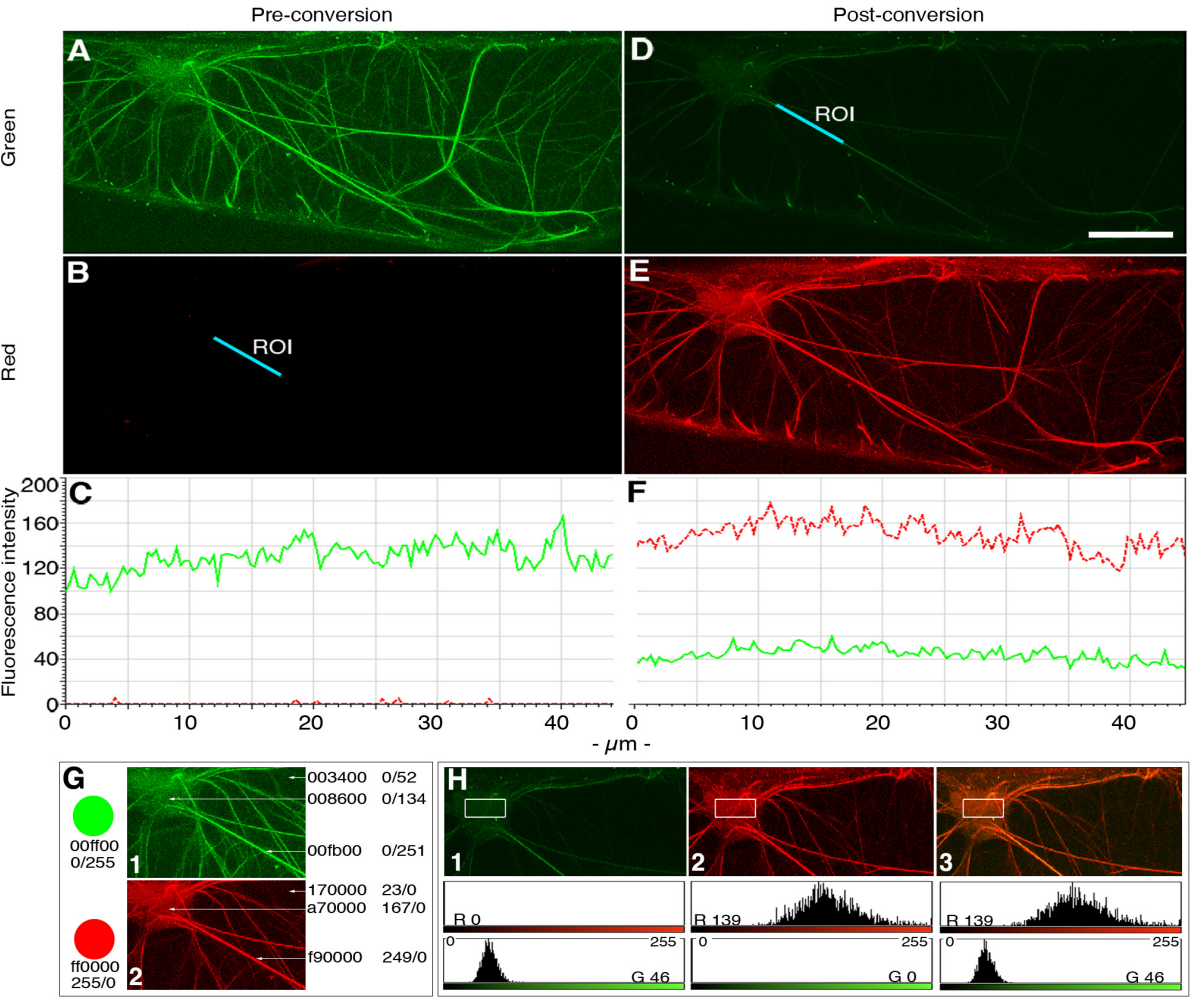
**Expression and photo-conversion of mEosFP::FABD-mTn following its transient expression in an onion epidermal cell.** Photo-conversion was carried out using a D-excitation filter (UV/Violet; Ex: BP355–425/Dich: 455/LP 470) on a Leica DM600B epi-fluorescent microscope. Pre- and post-conversion images were acquired using 488 and 543 nm laser lines. Images were acquired within 10 seconds after photo-conversion. A.B.C. Pre-conversion fluorescence status 'A' in the green fluorescence acquisition channel; 'B' in the red channel; C depicts the fluorescence intensity along a straight line ROI (panel B, D) for both channels. Note the very low level of fluorescence picked up by the Leica fluorescence quantification tool for the red channel as compared to the green channel. D.E.F. Post conversion fluorescence status for green ' D' and red 'E' channels. Color profile shows a complete inversion in the relationship between green and red fluorescence emissions along the selected ROI. Images D and E were captured within 10 seconds of photo-conversion. The fluorescence intensity scale is based on a 0–255 RGB colour code. Bar = 50 μm. G. An Adobe Photoshop-based approach to draw out information on colour quality and the relative red/green values in an internationally accepted colour code. Absolute green is 00ff00 or 0/255 while absolute red is ff0000 or 255/0. The 'eye-dropper' tool in Photoshop provides direct readouts of R/G values. Arrows point to examples of some readouts from cropped regions of A and E. H. An imageJ-based approach for creating colour histograms from an image. The red and green components in the rectangular ROI in panels 1–3 are depicted on a scale of 0–255 for each colour. Panel 1 and the histogram below it depict greenness within the image (cropped portion of panel D); Panel 2 (cropped portion of panel E) depicts redness in the same region after photo-conversion whereas panel 3 is a merge of panel 1 and 2 and accurately reflects the merged mean values obtained for them.

**Figure 3:**
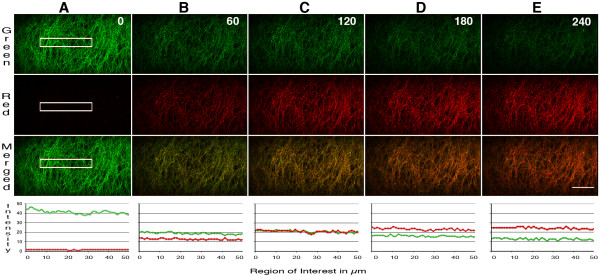
**Time lapse images on the xy axes of a region of an onion epidermal cell expressing mEosFP::FABD-mTn taken before photo-conversion and after 60 seconds exposition through a D-filter (UV/Violet; Ex: BP355–425/Dichr: 455/LP 470) using a 0.5 NA 20× lens, demonstrate the progressive enrichment of red fluorescent species over green protein molecules.** Accompanying traces for the ROI depicted in panel A confirm the changes observed visually. Whereas a significant jump in red fluorescence can be seen in Panel B already the amount of green fluorescent molecules is still higher, nearly equal fluorescence values appear in panel C and a significant increase is seen in red fluorescence in panels D and E. Size Bar = 25 μm.

The GFP-mTn probe [13] has been used successfully over the last decade for studying the F-actin organization during development of a variety of plant cell types. GFP in the original probe [13] was exchanged for mEosFP. For this XhoI and NaeI sites were introduced at the 5' and 3' of mEosFP before replacing the GFP for EosFP in an intermediate vector [13]. Transient expression of the fusion construct in onion epidermal cells and its visualization on a Leica TCS-SP5 confocal laser scanning microscope shows cells that fluoresced (Figure 2A) in the 500 to 525 nm (green channel) but not (Figure 2B, C) in the 585 to 680 nm collection band (red channel). The green fluorescent cells, visualized using a 40× water immersion lens (N.A. 1.25) on an epi-fluorescent microscope were illuminated directly for 60 seconds with UV/violet light obtained through a 'D' filter cube (Leica microsystems: Excitation filter BP 355–425; dichromatic mirror 455; suppression filter LP 470). This resulted in rapid loss of green fluorescence (Figure 2D) with a concomitant increase in red fluorescence (Figure 2E, F). It is known that transient expression methods such as the DNA-coated gold-particle bombardment employed by us result in variability between experiments and varying protein expression levels in different cells within a single experiment [32]. In our experiments the unequal protein expression levels were discernable as differing intensities of green fluorescence ranging from barely perceptible to strongly fluorescent. As a consequence the time required for photo-conversion of EosFP from green to red in different cells ranged from 1.5 to 4 minutes. Whereas these observations resulted from an innate limitation of transient expression techniques they highlighted the need for a careful estimation of photo-conversion time in different cell types. Adherence to a generalized photo-conversion time even in stable transgenic lines might result in non-converted proteins within a cell and lead to erroneous conclusions. Another factor that contributed to photo-activation time was the quality of the lens through which the activation light was transmitted. In general, as compared to the time taken when using a 20× lens (N.A. 0.5) the exposure time required for complete photo-conversion in a cell was reduced by half upon using a 40× water immersion lens (N.A. 1.25). Moreover, though photo-conversion of individual molecules of mEosFP::FABD-mTn can be portrayed as instantaneous, time-lapse confocal imaging of the F-actin mesh in a cell revealed that, in practical terms the conversion of all the molecules of the fusion protein within a cell can take a considerably longer time (Figure 3). During this time there is a progressive enrichment of red over green fluorescent molecules. As demonstrated through 5 time lapse images taken before (0-time point) and after every 60 seconds of photo-conversion (total 240 seconds of exposure) the fluorescence of F-actin strands in a cell progresses through intermediate shades of yellow and orange before becoming red (Figure 3A–E). Whereas looking at only the red and green channels suggests a clear difference after 120 seconds, the merged image is still yellow-orange. The accompanying fluorescence intensity graphs of the region shows a considerable overlap between the green and red fluorescent species and supports the visual yellow-merge in a quantitative manner. As reflected in the graphs subsequent exposure results in a further increase in red fluorescence (Figure 3D, E). This information is important when dealing with EosFP and similar photo-convertible proteins. For example, in situations where EosFP or similar proteins are being used to monitor organelle fusion a partial conversion can easily create a yellow colour. This can be misinterpreted as accumulation of green and red fluorescent species within an organelle and suggest organelle fusion. Since partial conversion due to protein under-expression, lens properties and target displacement away from the focus of the exciting light can create errors in interpretation of results it is imperative that for each cell type under consideration the time taken for full conversion to red should be determined accurately. EosFP does not appear to get photo-converted even after prolonged exposure to the 488 nm wavelength. However, the green fluorescent species does get photo-bleached. Thus prolonged exposure of a cell to blue light can have the effect of allowing an orange-red cell auto-fluorescence to be collected by the spectral detectors and create the illusion of photo-conversion. This can create an artifactual merged image where the red emission appears to dominate the emissions collected in the green channel. Care must be taken to observe a clear increase, and preferably an inversion in green-red spectral properties of the unconverted versus the photo-converted protein. A colour line-trace or histogram based approach to monitor fluorescence emission, its quantification and clear colour discrimination within the acquired images is necessary.

### Colour discrimination and quantification

Three different approaches were adopted for colour quantification and fluorescence data presentation. These included the use of proprietary software bundled with the Leica TCS SP5 confocal microscope (Live Data mode; Intensity quantification tool); use of the open source, public domain Java image processing program ImageJ , and a simple Adobe Photoshop based approach. Whereas the Leica TCS-SP5 software is user friendly it comes only as part of the confocal system. The imageJ program is freely downloadable, has been developed to match rigorous scientific standards, and is well accepted. The Adobe Photoshop colour coding is ICC compliant [29] and provides a useful addition to other methods for presenting data. The Leica TCS-SP5 intensity-plotting tool provides relative fluorescence intensities in different collection channels over a region of interest (ROI). Intensity plots along a free-form line (eg. Figure 1B, E, F), a straight line (eg. Figure 2C, D, F) and for a polygonal ROI (Figure 3) are presented. These line graphs adequately convey the relative fluorescence intensities of the green and red forms of mEosFP in a region of interest and highlight the shift from green to red. However, the question of complete conversion to red remains. This was sorted out by imageJ and Adobe Photoshop both of which use the RGB colour model. In this [29], colour is expressed as a numeric RGB triplet each component of which is an integer with a value ranging from zero to 255 (the range of 256 values offered by a single-8 bit byte). Thus Red is 256,0,0 and Green as 0,255,0. The international HTML standard colour representations in the Adobe Photoshop palette also provide a notation where each colour can take one of six values (eg. red-ff0000; yellow-ffff00; green-00ff00). For our images two reference spots depicting absolute green (00ff00 – 0/255) and absolute red (ff0000 – 255/0) were created on a separate layer using Adobe Photoshop (Figure 2G). Subsequently any point on a colour image acquired through epi-fluorescent or confocal microscopy could be selected using the 'eye-dropper' tool and a direct read out on RGB values. A demonstration of this method is shown in Figure 2G where panel 1 and panel 2 have been cropped from Figure 2A and Figure 2E and show pre- and post- conversion states of mEos::FABD-mTn, respectively. The colour values provided reflect R/G values at the end of each arrow and provide a real colour estimate in relation to an absolute red and green. Figure 2H (panels 1–3) presents channel specific histograms acquired using imageJ program. The histograms depict changes in green versus red colour spread over a 0–255 scale for each channel. Panel 3 is a merge of panels 1 and 2 and its histogram reflects this property.

The colour quantification approach provides direct measurement of the degree of red and green in an image. Through imaging of a number of independent samples it allows a statistical accuracy for hitherto purely empirical observations on actin organization.

### mEosFP::mTn in multicolour live visualization schemes

**Figure 4:**
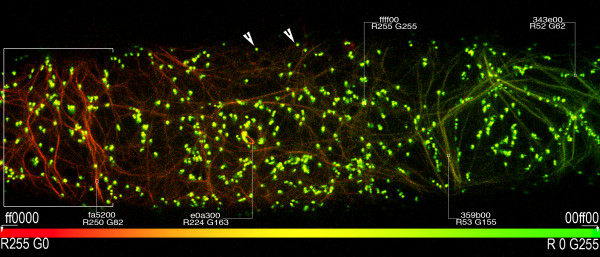
**Localized photo-activation of mEosFP::FABD-mTn along with a YFP-SKL marker targeted to peroxisomes demonstrates the feasibility of using the F-actin marker in simultaneous multicolour live-imaging schemes**. The left portion of the cell was photo-converted and exhibits a visible change in coloration. The ICC-compliant standard representation of colour coding has been followed. The colour bar thus extends between absolute red (R255 G0/ff0000) to absolute green (R0 G255/00ff00) and demonstrate the spread of colours in the image. Small regions of interest sampled using the eye-dropper tool in Adobe Photoshop provided direct read-outs of colour values from the image. Note that peroxisomes (arrowheads) highlighted using YFP-SKL maintain a median (R255 G255/ffff00) value.

Having established mEos::FABD-mTn as a photo-convertible probe for F-actin and the parameters for its quantification, its suitability for localized conversion and application in multicolour live-imaging schemes was tested. For achieving this the mEosFP::FABD-mTn was co-expressed transiently with a YFP-SKL marker that targets to peroxisomes [30]. A portion of each cell expressing both markers was illuminated with a ca~400 nm beam for 5 seconds. Subsequent acquisition of data in both green and red channels showed a clear green to red conversion of F-actin in the illuminated region (bracket: Figure 4) while the region of the cell farthest from the focus of illumination maintained green fluorescent F-actin (right side – Figure 4). The region in between showed partial conversion of mEosFP::FABD-mTn (mid-region-Figure 4). Whereas the partial colour conversion might be interpreted as a reflection of rapid actin dynamics we are confident that it has been produced through peripheral epi-fluorescent illumination rather than the spreading of photo-converted protein. Nevertheless, an analysis of actin dynamics is possible using small, rigidly defined confocal ROIs with minimal peripheral illumination. An analysis of regional actin dynamics using EosFP-mTn is underway and will be reported independently.

In this experiment peroxisomes, highlighted in yellow (arrowheads – Figure 4) could be easily co-visualized with both forms of mEosFP labelled F-actin without the need for a more specific collection channel. While other FPs are still being evaluated, this experiment clearly demonstrates that mEosFP::FABD-mTn can be used easily in a multicolour live imaging scheme with YFP. Nevertheless, while the co-visualization scheme works very well with morphologically discrete and motile organelle like the peroxisomes, given the points on partial conversion raised earlier, the co-visualization of similar elements like actin-microfilaments and cortical microtubules should be approached with caution.

## Conclusion

A number of probes have been created for visualizing F-actin organization in plant cells. None of them appears to be perfect and different research groups have their own preference and protocols for dealing with these probes. The mEosFP::FABD-mTn probe is based on the well established probe GFP::mTn [13] and retains all the properties associated with the parent probe. It can be used for visualizing F-actin organization, actin dynamics and interactions with other organelles. However, unlike GFP::mTn [13] and GFP::FIM1-ABD2 [14] the new probe with a monomeric FP offers both global and localized colour discrimination in a cell through its easy and rapid photo-convertibility. In addition the sub-cellular colour conversion is amenable to dissection and quantification using well developed colour quantification tools that conform to international colour coding standards. Though our assessment of the long-term effects of mEosFP::FABD-mTn on actin organization and cell morphogenesis in transgenic plants is underway, mEosFP::FABD-mTn is potentially the most versatile probe for observing F-actin in living plant cells.

## Methods

### Molecular techniques

Standard molecular cloning techniques were followed [31]. Monomeric EosFP in a pcDNA3-Flag1 vector [27] was cut at KpnI/ApaI sites. The fragment was placed between a CaMV35S promoter and a 'nos' terminator sequence that had been introduced in a pCAMBIA-1300 vector  to create mEosFP-cytosolic. For creating the F-actin targeting fusion construct, first GFP(XhoI-NaeI)::mTn (NaeI-SpeI) was cut as an XhoI-SpeI fragment from the pBA005 vector [13] and reintroduced into an intermediate vector carrying a CaMV35S promoter and a nos terminator. mEosFP was PCR amplified using primers JM 817A CCACGctcgagATGAGTGCGATTAAGCCA and JM 810 – ATTATTgccggcTCGTCTGGCATTGTCAG to introduce XhoI and NaeI sites at its N- and C- terminus respectively, while removing the stop codon. The GFP in the XhoI-NaeI sites was replaced by the PCR amplified mEosFP fragment to yield the mEosFP (XhoI/NaeI):: FABD-mTn (NaeI/SpeI) fusion used in this study. The YFP-SKL construct targeting to peroxisomes has been described earlier [30].

### Transient expression in plant cells

Plasmid DNA obtained through routine DNA mini-preparations (GeneJET plasmid miniprep kit) was used for transient expression studies.

Transient expression of mEosFP-cytosolic, mEosFP::FABD-mTn and of YFP-SKL in onion epidermal cells was carried out through 1 μm diameter gold-particles coated with ca. 2.5 μg DNA and bombarded using a biolistic particle delivery system (Biorad PDS-1000/He). Manufacturers' instructions were followed for this procedure. Co-expression of mEosFP::FABD-mTn and YFP-SKL was achieved by mixing the two DNAs (ca. 1.25 μg each) before coating the 1 μm gold particles. Protein expression was assessed as fluorescence between 16 and 20 hours after shooting using an epi-fluorescent microscope.

### Microscopy and imaging

Upright epi-fluorescent microscopes Nikon eclipse 80i and a Leica DM6000B were used. Filter sets from Chroma  used on the Nikon microscope were: Endow GFP-LP filter set 41018 (Ex: HQ 470/40X; Dichr: Q495LP; Em: HQ500LP); TRITC filter set 41002c (Ex: HQ545/30X; Dichr: Q570LP; Em: HQ 620/60m); FITC/Texas Red filter set 51006 (Ex: 51006X; Dichr: 51006bs; Em: HQ535/30m); DAPI/Hoechst/AMCA filter set: 31000V2 (D 350/50X; CLP 400; D 460/50m). Glass filter cubes used on the Leica DM6000-B microscope were: filter cube D (UV/Violet; Ex: BP355–425/Dichr: 455/LP 470), I3 (Ex:BP 450–490; Dichr 510/Em:LP515); N2.1 (BP 515–560; Dichr: 580; Em. LP590). The Leica microscope forms a component of the TCS-SP5 confocal imaging system, which utilizes an Ar laser: 488 nm and a HeNe laser 543 nm. Photo-conversion on the confocal microscope was done using epi-fluorescent lighting through filter cube D. Partial photo-conversion was achieved by closing down the iris or moving the stage so that only a small part of the cell was exposed to the beam. For Figures 1, 2 and 3 the entire cell was exposed with the focal point of the beam being maintained around the cell centre. For Figure 4 the area illuminated by the 405 nm beam is depicted.

For imaging the 488 nm laser line was used at ca. 10% of its power while the weak 543 nm laser was used at 98% power. Increasing 488 nm laser power affected bleed through of fluorescence into the red channel and also increased photo-bleaching during repeated scans. Images were acquired within 10 seconds after photo-conversion in a 1024 × 512 pixel format. Time lapse between scans in xyt mode was 1.37 seconds whereas in the xyz mode sequential images had 1 μm (Z axis) between them. Emission wavelength collection on the spectrophotometric Leica confocal was maintained between 500 to 525 nm (green channel) and 585 to 680 nm (red channel).

### Post-acquisition image processing

Images acquired using the Leica confocal were processed directly using the fluorescence intensity quantification tools in different regions of interest (ROI). All images were cropped and processed for brightness/contrast as complete montages using Adobe Photoshop CS3. The layer function in Photoshop was used to introduce text, ROIs and colour overlays. These images were subjected to analysis using the eye-dropper tool in Adobe Photoshop (eg. Figure 2G), and the polygon and line selection tools in ImageJ. Histograms (eg. Figure 2H) in imageJ were created using colour histogram function in the 'analyze' tool palette. The 0 – 255 integer scale (256 values) used for 8-bit colour coding has been used in these histograms with red being defined as 255/0 and green 0/255. The blue channel in the resultant histograms was at constant value of '0' and was cropped out. For colour coding the ICC-compliant RGB triplet code for true colours and HTML based web applications code [29] followed by Adobe Photoshop has been used.

## Competing interests

The authors declare that they have no competing interests.

## Authors' contributions

MS created the mEOsFP-FABD-mTn probe, carried out its characterization and created the figures, AMS and DJ contributed equally in carrying out the experiments using mEos-Cyto and YFP-SKL. JM conceived and co-ordinated the experiments and drafted the manuscript. JDB edited the manuscript. All authors read and approved the final manuscript.
